# Clinical Significance of Germline Cancer Predisposing Variants in Unselected Patients with Pancreatic Adenocarcinoma

**DOI:** 10.3390/cancers13020198

**Published:** 2021-01-08

**Authors:** Elena Fountzilas, Alexia Eliades, Georgia-Angeliki Koliou, Achilleas Achilleos, Charalambos Loizides, Kyriakos Tsangaras, Dimitrios Pectasides, Joseph Sgouros, Pavlos Papakostas, Grigorios Rallis, Amanda Psyrri, Christos Papadimitriou, Georgios Oikonomopoulos, Konstantinos Ferentinos, Anna Koumarianou, George Zarkavelis, Christos Dervenis, Gerasimos Aravantinos, Dimitrios Bafaloukos, Paris Kosmidis, George Papaxoinis, Maria Theochari, Ioannis Varthalitis, Nikolaos Kentepozidis, Georgios Rigakos, Zacharenia Saridaki, Adamantia Nikolaidi, Athina Christopoulou, Florentia Fostira, Epaminontas Samantas, Elena Kypri, Marios Ioannides, George Koumbaris, George Fountzilas, Philippos C. Patsalis

**Affiliations:** 1 Second Department of Medical Oncology, Euromedica General Clinic of Thessaloniki, 54645 Thessaloniki, Greece; elenafou@gmail.com; 2 European University Cyprus, 1516 Engomi, Cyprus; 3 NIPD Genetics Limited, 2409 Nicosia, Cyprus; a.eliades@nipd.com (A.E.); a.achilleos@nipd.com (A.A.); c.loizides@nipd.com (C.L.); k.tsangaras@nipd.com (K.T.); e.kypri@nipd.com (E.K.); m.ioannides@nipd.com (M.I.); g.koumbaris@nipd.com (G.K.); 4 Section of Biostatistics, Hellenic Cooperative Oncology Group, Data Office, 11526 Athens, Greece; g_koliou@hecog.ondsl.gr; 5 Oncology Section, Second Department of Internal Medicine, Hippokration Hospital, 11527 Athens, Greece; pectasid@otenet.gr; 6 Third Department of Medical Oncology, Agii Anargiri Cancer Hospital, 14564 Athens, Greece; josephsgouros@yahoo.co.uk (J.S.); epsam@otenet.gr (E.S.); 7 Oncology Unit, Hippokration Hospital, 11527 Athens, Greece; p.papakostas@gmail.com; 8 Department of Medical Oncology, Papageorgiou Hospital, Aristotle University of Thessaloniki, School of Health Sciences, Faculty of Medicine, 56429 Thessaloniki, Greece; gsrallis@auth.gr; 9 Section of Medical Oncology, Department of Internal Medicine, Attikon University Hospital, Faculty of Medicine, National and Kapodistrian University of Athens School of Medicine, 12462 Athens, Greece; psyrri237@yahoo.com; 10 Oncology Unit, Aretaieion Hospital, National and Kapodistrian University of Athens School of Medicine, 11528 Athens, Greece; chr_papadim@yahoo.gr; 11 Second Department of Medical Oncology, Metropolitan Hospital, 18547 Piraeus, Greece; Goik77@yahoo.com; 12 Department of Radiation Oncology, German Oncology Center, European University Cyprus, 4108 Limassol, Cyprus; Konstantinos.Ferentinos@goc.com.cy (K.F.); fountzil@auth.gr (G.F.); 13 Fourth Department of Internal Medicine, Hematology-Oncology Unit, Attikon University Hospital, National and Kapodistrian University of Athens, 12462 Athens, Greece; akoumari@yahoo.com; 14 Department of Medical Oncology, University Hospital of Ioannina, Medical School, 45500 Ioannina, Greece; gzarkavelis@outlook.com; 15 First Department of Surgery, General Hospital Konstantopouleio Agia Olga, 14233 Athens, Greece; dervenis.christos@ucy.ac.cy; 16 Second Department of Medical Oncology, Agii Anargiri Cancer Hospital, 14564 Athens, Greece; garavantinos@yahoo.gr; 17 First Department of Medical Oncology, Metropolitan Hospital, 18547 Piraeus, Greece; dimmp@otenet.gr; 18 Second Department of Medical Oncology, Hygeia Hospital, 15123 Athens, Greece; p.kosmidi@hygeia.gr; 19 Second Department of Internal Medicine, Agios Savvas Cancer Hospital, 11522 Athens, Greece; georgexoinis@gmail.com; 20 First Department of Medicine, Laiko General Hospital, National and Kapodistrian University of Athens School of Medicine, 11528 Athens, Greece; secretaryapk@med.uoa.gr; 21 Oncology Department, General Hospital of Chania, 73300 Crete, Greece; vari49@gmail.com; 22 Department of Medical Oncology, 251 Airforce General Hospital, 11525 Athens, Greece; kentenik@hotmail.com; 23 Third Department of Medical Oncology, Hygeia Hospital, 15123 Athens, Greece; grigakos@oncologists.gr; 24 Asklepios Oncology Department, 71303 Heraklion, Crete, Greece; zeniasar@gmail.com; 25 Oncology Clinic, Mitera Hospital, 15123 Athens, Greece; mantonikolaidi@gmail.com; 26 Medical Oncology Unit, S. Andrew Hospital, 26335 Patras, Greece; athinachristo@hotmail.com; 27 Molecular Diagnostics Laboratory, InRASTES, National Centre for Scientific Research “Demokritos”, 15341 Athens, Greece; florentia_fostira@hotmail.com; 28 Laboratory of Molecular Oncology, Hellenic Foundation for Cancer Research/Aristotle University of Thessaloniki, 54006 Thessaloniki, Greece; 29 Aristotle University of Thessaloniki, 54124 Thessaloniki, Greece; 30 Medical School, University of Nicosia, 2417 Nicosia, Cyprus

**Keywords:** BRCA2, inherited, overall survival, predictive, prognostic

## Abstract

**Simple Summary:**

Data on the prognostic and predictive value of germline cancer predisposing pathogenic/likely pathogenic variants (P/LPVs) in patients with pancreatic ductal adenocarcinoma is limited. This research was a study of germline testing of 62 cancer susceptibility genes in 549 unselected patients with pancreatic cancer. We reported a significant proportion of European patients with pancreatic cancer carrying P/LPVs in clinically significant genes, irrespectively of age, family history or disease stage. Importantly, P/LPVs were identified in pancreatic cancer-associated genes and in homologous recombination repair genes in 4.0% and 7.7% of patients, respectively. The presence of any P/LPVs was associated with improved overall survival univariately; however, it did not retain its independent prognostic significance in multivariate analysis. The presence of P/LPVs in homologous recombination repair genes did not predict benefit from platinum-based treatment. These results should be prospectively validated through universal genetic testing of patients with pancreatic cancer, taking into consideration the administration of newer treatments.

**Abstract:**

Our aim was to determine the prevalence, prognostic and predictive role of germline pathogenic/likely pathogenic variants (P/LPVs) in cancer predisposing genes in patients with pancreatic ductal adenocarcinoma (PDAC). Germline testing of 62 cancer susceptibility genes was performed on unselected patients diagnosed from 02/2003 to 01/2020 with PDAC, treated at Hellenic Cooperative Oncology Group (HeCOG)-affiliated Centers. The main endpoints were prevalence of P/LPVs and overall survival (OS). P/LPVs in PDAC-associated and homologous recombination repair (HRR) genes were identified in 22 (4.0%) and 42 (7.7%) of 549 patients, respectively. P/LPVs were identified in 16 genes, including *ATM* (11, 2.0%) and *BRCA2* (6, 1.1%), while 19 patients (3.5%) were heterozygotes for *MUTYH* P/LPVs and 9 (1.6%) carried the low-risk allele, *CHEK2* p.(Ile157Thr). Patients carrying P/LPVs had improved OS compared to non-carriers (22.6 vs. 13.9 months, *p* = 0.006). In multivariate analysis, there was a trend for improved OS in P/LPV carriers (*p* = 0.063). The interaction term between platinum exposure and mutational status of HRR genes was not significant (*p*-value = 0.35). A significant proportion of patients with PDAC carries clinically relevant germline P/LPVs, irrespectively of age, family history or disease stage. The predictive role of these P/LPVs has yet to be defined. ClinicalTrials.gov Identifier: NCT03982446.

## 1. Introduction 

Germline pathogenic/likely pathogenic variants (P/LPVs) DNA repair genes, including *BRCA1*, *BRCA2*, *ATM*, *PALB2,* and mismatch repair (MMR) genes and *EPCAM* has been associated with increased risk for pancreatic adenocarcinoma (PDAC) and other tumors [1,2,3,4,5,6]. The presence of P/LPVs in these genes has several implications for patients with PDAC and their healthy relatives, who might be carriers of the respective variants. First, the administration of innovative treatments has been shown to be associated with improved clinical outcomes in patients with PDAC with selected molecular alterations [7,8,9,10]. Importantly, patients and healthy individuals who carry cancer predisposing P/LPVs may benefit from intensive screening protocols, risk-reducing surgeries and/or chemoprevention strategies. Therefore, the National Comprehensive Cancer Network (NCCN) currently recommends genetic counseling and genetic testing to all patients with PDAC as well as to first-degree relatives of patients with PDAC [11]. 

Recent studies have demonstrated that 3.8–20.7% of patients with PDAC carry germline P/LPVs in cancer predisposing genes [6,12,13,14,15,16,17,18,19,20,21,22,23,24]. Through these studies, it has been highlighted that family history, disease stage or age at diagnosis are not always predictors of an underlying genetic factor, while P/LPVs status is often associated with phenotypic heterogeneity. Most studies focused mainly on the prevalence of P/LPVs by using, often limited, gene panels in heterogenic patient populations, while few studies investigated the prognostic significance of PVs [9,12].

The aim of this study was to determine the prevalence of germline P/LPVs in cancer predisposing genes, in unselected patients with PDAC, treated at Departments of Oncology affiliated with the Hellenic Cooperative Oncology Group (HeCOG), with the use of an extensive multigene panel that tests for 62 genes implicated in cancer susceptibility with the concurrent detection single nucleotide variants (SNVs), insertions and deletions (Indels), as well as exon-level copy number variants (CNVs). To our knowledge, this is the first European study of PDAC patients of its size in which the prevalence of germline P/LPV and their prognostic and predictive role are investigated in a large number of genes. 

## 2. Results

### 2.1. Patient Characteristics and P/LPV Prevalence 

In total, 549 patients diagnosed with PDAC were included in the current analysis. Overall, 99.1% of them (*n* = 544) were treated in Greek medical departments of both public (456 patients; 83.1% of total population) and private hospitals, while 5 patients (0.9%) were recruited in a private oncology center in Cyprus. Median age at diagnosis was 65 years (range 34−86), and 278 (50.6%) patients were men. Of 549 patients, 62 (11.3%) carried P/LPVs in any of the cancer predisposing genes tested and 22 (4.0%) carried P/LPVs in genes known to be associated with pancreatic cancer. Family history of cancer was reported more commonly in patients carrying P/LPVs (*n*= 26, 44.1%) compared to non-carriers (*n* = 115, 27.9%) (chi-square *p* = 0.011). Of 59 patients with P/LPVs and available data for family history, 8 (13.6%) reported a family history of PDAC and 14 (23.7%) of other tumor types (tumor type was not available in 4 patients). Patient detailed clinicopathological characteristics are summarized in Table 1.

**Figure 1:**
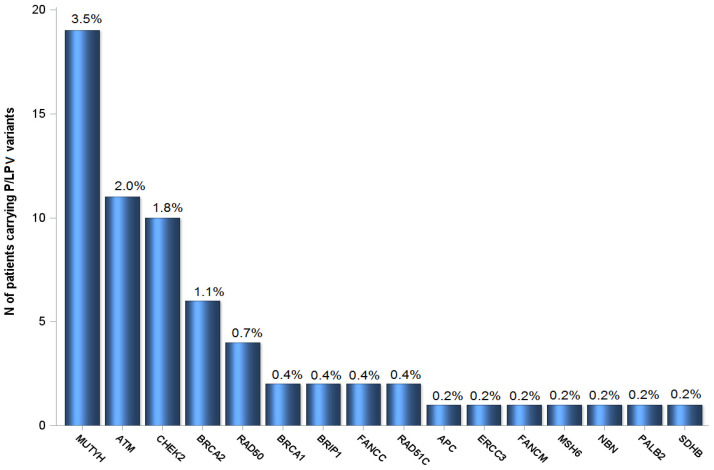
Prevalence of P/LPVs in 549 patients of the study. Single nucleotide variants (SNV), insertions/deletions (Indels) and copy number variant (CNV) detection was performed for the following genes: *APC*, *ATM*, *BAP1*, *BARD1*, *BMPR1A*, *BRCA1*, *BRCA2*, *BRIP1*, *CDH1*, *CDK4*, *CDKN2A*, (*CDKN2A*^p16(INK4A)^, *CDKN2A*^p14(ARF)^), *CHEK2*, *DDB2*, *DICER1*, *EPCAM*, *ERCC1*, *ERCC2*, *ERCC3*, *ERCC4*, *ERCC5*, *FANCA*, *FANCB*, *FANCC*, *FANCD2*, *FANCE*, *FANCF*, *FANCG*, *FANCI*, *FANCL*, *FANCM*, *GREM1*, *HOXB13*, *MEN1*, *MLH1*, *MRE11*, *MSH2*, *MSH6*, *MUTYH*, *NBN*, *PALB2*, *PMS2*, *POLD1*, *POLE*, *POLH*, *PTEN*, *RAD50*, *RAD51C*, *RAD51D*, *RB1*, *RET*, *SDHAF2*, *SDHB*, *SDHC*, *SDHD*, *SLX4*, *SMAD4*, *SMARCA4*, *STK11*, *TP53*, *VHL*, *XPA*, *XPC*. Overall, 62 patients (11.3%) carried ≥1 P/LPV in at least 1 of 16 genes. Three patients carried two P/LPVs; two carried P/LPVs in *CHEK2* and *MUTYH* and one in *BRCA1* and *MUTYH*. The rest of the patients (59) had one P/LPV detected. The percentage of patients carrying a P/LPV in each gene is shown on top of each bar.

Genes with P/LPVs in ≥2 patients were *ATM* (*n* = 11, 2.0%), *BRCA2* (*n* = 6, 1.1%), *RAD50* (*n* = 4, 0.7%), *BRCA1* (*n* = 2, 0.4%), *BRIP1* (*n* = 2, 0.4%), *FANCC* (*n* = 2, 0.4%) and *RAD51C* (*n* = 2, 0.4%). In addition, 19 patients (3.5%) carried P/LPVs in *MUTYH,* while 9 (1.6%) carried the low-risk allele, *CHEK2* p.(Ile157Thr); none of them had previous history of colorectal or breast cancer, respectively. The distribution of P/LPVs is presented in Figure 1. Notably, P/LPVs in genes participating in the homologous recombination repair (HRR) system (*CHEK2* p.(Ile157Thr) excluded) accounted for 7.7% (42 patients). Interestingly, in one patient, a PV was identified in *SDHB*, a gene with no previous association with PDAC. In addition, 2 CNVs (3-exon deletion in *ATM* and 2-exon deletion in *FANCC*) were identified in 2 patients, accounting for 3.2% of the total number of P/LPVs identified. All P/LPVs are reported per patient in Table S1.

Of note, most frequent P/LPVs in low-risk genes (*MUTYH*, *RAD50* and *CHEK2*) were compared to population frequencies (gnomAD database, comparing against the population with the highest frequency for the given P/LPVs, details in Methods). Two P/LPVs were found in higher frequency in our patient population compared to healthy population (control): *MUTYH* p.Arg245His (0.00820 vs. 0.00117, *p* = 0.002) and *MUTYH* p.Glu480del (0.00273 vs. 0.00061, *p* = 0.049) (Table S2). 

### 2.2. Clinical Outcomes 

**Figure 2:**
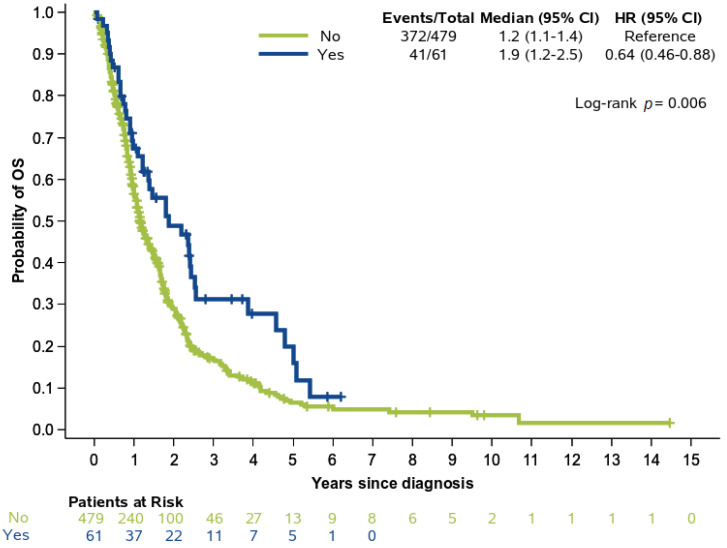
Overall survival (OS) in patients with and without P/LPVs; analysis was performed in 540 patients with available outcome data.

Within a median follow-up of 47.31 months (95% CI 34.75−64.10), a total of 413 (76.5%) deaths were reported. The median OS for the entire population with available survival data (*N* = 540) was 14.52 months (95% CI 13.08−16.52). Patients with P/LPVs had improved OS compared to non-carriers (22.62 months (95%CI 14.69−30.49) vs. 13.90 months (95% CI 12.82−16.23), log-rank *p* = 0.006) (Figure 2). The favorable prognostic significance of P/LPVs with respect to OS was underlined in the group of patients with early-stage disease (HR 0.47, 95% CI 0.26−0.84, *p* = 0.011), while significance was not reached among patients with advanced stage pancreatic cancer (HR 0.91, 95% CI 0.61−1.34, *p* = 0.62) even though the direction of the hazard ratio was retained. Upon multivariate analysis, there was only a trend for improved OS in P/LPV carriers (Wald’s overall *p* = 0.063) observed in the group of patients who had not received platinum-based chemotherapy (HR 0.65, 95% CI 0.43−1.05), whereas no difference was detected among platinum-treated patients (HR 0.77, 95% CI 0.47−1.23) (Table 2). The interaction term between the presence of P/LPVs and platinum treatment did not show a significant predictive benefit in the multivariate model (interaction *p* = 0.60), while, as expected, early stage contributed to significantly longer OS as compared to advanced stage of disease (HR 0.37, 95% CI 0.29−0.46, *p* < 0.001). The therapeutic effect of platinum exposure was also significant overall in the multivariate analysis (*p* = 0.002). 

When excluding patients carrying only *MUTYH* P/LPVs and *CHEK2* p.Ile157Thr (*N* = 25), a trend for improved OS was identified for patients with P/LPVs compared to non-carriers overall (21.61 months, 95%CI 11.34−30.69 vs. 13.90 months, 95% CI 12.82−16.23, respectively, log-rank *p* = 0.068). In addition, a trend towards longer OS was detected for patients with P/LPVs and early-stage disease (HR 0.52, 95% CI 0.25−1.06, *p* = 0.073), whereas no prognostic significance was found in patients with advanced disease (HR 1.10, 95% CI 0.65−1.85, *p* = 0.72). The interaction term between platinum exposure and mutational status of HRR genes (excluding *CHEK2* p.Ile157Thr) was not significant either in the entire cohort (interaction *p*-value = 0.35) or among patients with early (interaction *p*-value = 0.42) or advanced disease (interaction *p*-value = 0.50). The prognostic significance of P/LPVs in PDAC-associated and HRR genes are shown in Table 3.

Among patients who received platinum-based therapy at any line of treatment, only a trend towards improved OS was identified for those who carried P/LPVs in HRR genes compared to non-carriers (HR 0.52, 95% CI 0.25−1.06, *p* = 0.074) (Table 3). OS was similar in patients who did not receive platinum agents, regardless of HRR P/LPV status (HR 0.86, 95% CI 0.48−1.55, *p* = 0.62). P/LPVs in PDAC-associated genes showed favorable prognostic significance univariately only among patients treated with platinum-based therapy (HR 0.34, 95%CI 0.12−0.92, *p* = 0.033). The presence of P/LPVs in HRR associated genes was not found to be prognostic for OS either among patients with early (HR 0.61, 95% CI 0.28−1.30, *p* = 0.20) or those with advanced stage (HR 1.09, 95% CI 0.63−1.86, *p* = 0.77). 

## 3. Discussion

In this observational study, germline testing of 62 cancer predisposing genes was performed in a cohort of 549 patients with PDAC. To our knowledge, this is the only European study that tests the presence of both sequence variants (SNVs and Indels) as well as CNVs in a large number of cancer-predisposing genes, providing a comprehensive understanding of the prevalence of P/LPVs in patients with PDAC. Our study revealed P/LPVs in 4.0% and 7.7% of patients, in PDAC-associated genes and HRR genes, respectively. Importantly, patients were not preselected for age, stage or family history of cancer, highlighting the unbiased character of our study, as these factors are not always predictors of an underlying genetic factor. Moreover, association of germline testing results, clinicopathologic parameters and outcome data was performed.

Similar to previous studies, our study has identified P/LPVs in 11.29% of patients [12,20,25]. Investigators have reported significantly different proportions of patients with PDAC carrying P/LPVs in cancer predisposing genes, ranging from 3.8% to 21%. This difference might be attributed to the different number of genes interrogated in each study [15,16], inclusion of high-risk carriers (Ashkenazi ancestry) [19,20], preselection of patients for positive family history [13] and focus on specific patient populations [16]. P/LPVs might also be random findings that are not associated with increased risk for PDAC. For instance, the number of patients carrying *MUTYH* P/LPVs or the *CHEK2* p.(Ile157Thr) might reflect the expected population frequency. Nevertheless, other investigators have previously reported the presence of P/LPVs in these genes in patients with PDAC [13,19,23,26] and, therefore, further investigation of their role is warranted. While the investigation of the presence of polyps in these patients would be clinically relevant, no data was available. Of note, in this study, a PV in SDHB was identified in a patient with PDAC with no prior history. However, detailed family history was not recorded for this patient to assess consistency with the features of the respective syndrome. The clinical significance of SDHB, a gene that has not previously been associated with PDAC predisposition, remains to be shown in future studies. Of note, our study has not identified any patient harboring TP53 P/LPV variants. Recent studies have estimated the prevalence of TP53 P/LPVs between 0.2% and 1.34% [6,16,27,28]. The heterogeneity observed regarding TP53 P/LPV prevalence might be attributed to the different study design (i.e patient cohort size, selection bias, ethnicity). SMAD4 P/LPVs have not been identified in our study, in accordance with the findings in recent studies [6,16,27,28]. Although molecular studies in sporadic pancreatic cancer have identified somatic mutations in TP53 and SMAD4 in high frequency, our study as well as other germline pancreatic cancer predisposition studies outlined here show that the hereditary contribution of these genes is rare. All P/LPVs identified in our study are heterozygous variants. This is in accordance with the mode of inheritance for these genes regarding their association with pancreatic cancer susceptibility [29].

While the presence of any P/LPV was associated with improved OS, in multivariate analysis it did not retain its prognostic significance, showing only a trend for improved OS in P/LPV carriers. In line with our findings, a previous study of 615 prospectively consented patients who underwent germline testing for 410–468 genes showed no difference in OS between patients with and without germline P/LPVs, either in the total population or in subgroup analysis (metastatic patients, carriers vs noncarriers of *BRCA* PVs or patients with and without tumor loss of heterozygosity of *BRCA* PVs) [19]. Another study similarly demonstrated that there was no difference in prognosis among carriers of P/LPVs and noncarriers [16]. However, the median OS of patients carrying P/LPVs in DNA damage repair genes was significantly longer compared to patients without such P/LPVs. On the contrary, a different study has shown that the presence of P/LPVs conferred a better prognosis to patients with PDAC [23]. Improved OS appeared to be associated primarily with P/LPVs in DNA damage repair genes. 

In our study, P/LPVs in HRR genes, excluding CHEK2 p.(Ile157Thr), were identified in 7.7% patients. This proportion is significant, since these patients might benefit from innovative treatments, such as PARP inhibitors or from platinum agents. Indeed, the addition of olaparib as maintenance treatment for patients with PDAC carrying germline P/LPVs *BRCA1/2* led to longer PFS compared to placebo [7]. However, a recent study showed that in pretreated patients with advanced breast cancer, responses to olaparib were gene specific [30]. No patient with *CHEK2* or *ATM* P/LPVs responded to treatment with PARP inhibitor. Therefore, the role of PARP inhibitors or platinum agents needs to be prospectively validated in patients with PDAC carrying HRR P/LPVs in different genes.

The predictive role of P/LPVs in DNA damage repair genes is being evaluated in several tumor types. In our study, we did not observe any statistically significant benefit from the administration of platinum-based therapy in patients carrying P/LPVs in HRR genes. This lack of benefit in our study might be attributed to inclusion of platinum agents administered at any line of treatment and high heterogeneity of treatment regimens. Importantly, to our knowledge, none of our patients received treatment with poly ADP-ribose polymerase (PARP) inhibitors or immunotherapeutic agents during the study period. We did, however, observe improved clinical outcomes in patients treated with platinum-based therapy carrying P/LPVs in PDAC-associated genes. Due to the small number of patients with the event of interest noted in this subgroup, however, this result should be interpreted with caution until further validated in larger cohorts. Other investigators who tested a similar list of HRR genes (*ATM*, *BRCA1*, *BRCA2*, *BRIP1*, *CHEK2*, *NBN*, *PALB2*, *RAD50*, *RAD51C* and *RAD51D*) also reported similar OS between patients with PVs in DNA damage repair genes who received oxaliplatin-based therapy and those who did not [23]. In contrast, other studies have shown clinical benefit from treatment with platinum agents and/or PARP inhibitors in selected patients with diverse tumor types and importantly in patients with pancreatic cancer [7,8,9]. Investigators have reported that patients with PDAC and somatic mutations in DNA damage repair genes who received platinum-based therapy had improved OS compared to patients without such mutations [31]. In another study, patients with germline PVs in *BRCA1*, *BRCA2* or *PALB2* had higher objective response rates when treated with platinum regimens compared to patients who did not carry germline PVs, although this study tested fewer HRR genes compared to our study [9]. The different outcomes amongst all these studies can be attributed to the different number of patients and number of HRR genes tested as well as the different setting of genetic testing (somatic versus germline). These differences additionally highlight the importance of prospective examination of the prognostic and predictive significance of germline P/LPVs in large series of patients with PDAC. 

We acknowledge that our study has certain limitations. First is the retrospective nature of the study. Second is the inclusion of only one endpoint. Additionally, this was an observational study and detailed family history was not obtained for some patients. Furthermore, tumor biopsy samples were not subjected to genetic testing. As such, loss of heterozygosity, which could shed some light into possible association causative role of the P/LPVs on PDAC, was not evaluated in the tumors. Finally, the relatively small number of patients with P/LPVs limits analysis in patient subgroups, which would demonstrate critical associations. 

## 4. Materials and Methods

### 4.1. Study Population 

Genomic DNA was retrospectively and prospectively (since May 2019) collected from patients with PDAC, who were unselected for family history, stage of disease, or age at diagnosis and were diagnosed from 02/2003 to 01/2020. Patients were recruited in 24 HeCOG-affiliated Departments of Oncology; 23 located in Greece (16 in Athens) and 1 in Cyprus. Written informed consent was obtained from all patients for the use of their biological material and their participation in research studies. Patient clinicopathologic characteristics, family history and outcome data were retrieved from medical records, following regulations of the Bioethics Committees of participating institutions. The study was approved by the Institutional Review Board of “Agii Anargiri” Cancer Hospital (541/06.05.2019). The trial was registered (NCT03982446). 

### 4.2. Multigene Panel Testing 

Next-generation sequencing was performed in a Clinical Laboratory Improvement Amendments (CLIA)-certified laboratory, using a panel of 62 cancer susceptibility genes (PreSENTIA™ pan-cancer gene panel, NIPD Genetics™), detecting SNVs, Indels and CNVs (Supplementary methods and Figure S1). Targeted genomic loci were captured using an in-solution hybridization method (NIPD Genetics™).

### 4.3. Bioinformatics and Data Analysis

Sequence data were de-multiplexed and aligned to the human genome built (hg19) using BWA-MEM to generate alignment (BAM) files. Variant calling were performed following GATK best practices workflow [32]. Custom built bioinformatics tools were used for CNV calling. Classification and interpretation of variants was performed according to established guidelines and were in line with ClinVar database [33,34]. Detailed methodology is described in the Supplement.

### 4.4. Statistical Analysis 

Patient characteristics and P/LPV frequencies were summarized using descriptive statistics and compared with the chi-square/Fisher’s exact (for categorical variables) and Wilcoxon rank-sum test (for continuous variables). Overall survival (OS) was measured from the date of initial diagnosis until the date of death (from any cause) or last contact and was assessed in 540 patients (98.4%) with available survival data. Survival curves were estimated using the Kaplan-Meier method and compared between groups by the two-sided log-rank test. The prognostic significance of P/LPVs was evaluated using Hazard Ratios (HR) and 95% Confidence Intervals (CI) estimated by univariate Cox regression analysis in the entire cohort with available data (*N* = 540). Additionally, a multivariate Cox model was applied in the entire cohort including the presence of P/LPVs (prognostic term), stage of disease, platinum exposure (treatment term) and the interaction between platinum exposure and the presence of P/LPVs (predictive term).

We further evaluated the prognostic significance of P/LPVs in PDAC- (*APC*, *ATM*, *BRCA1*, *BRCA2*, *MSH6* and *PALB2*) and HRR- (*ATM*, *BRCA1*, *BRCA2*, *BRIP1*, *FANCC*, *CHEK2*, *FANCM*, *NBN*, *PALB2*, *RAD50*, *RAD51C*) associated genes, upon exclusion of patients who carried only *MUTYH* P/LPVs and *CHEK2* p.Ile157Thr, (*N* = 515) and in the subgroups of patients defined by treatment type (platinum-based chemotherapy yes vs. no). The reference group for comparisons was the group of patients without P/LPVs, including those with no P/LPVs in each of the aforementioned gene categories and patients with no P/LPVs at all. The predictive value of P/LPVs for platinum-based therapy was estimated univariately by interactions tests between each gene category and platinum exposure (yes vs. no). Departures from the proportional hazards assumption were assessed using time dependent covariates. All tests were two-sided and significance was set at 0.05. Analysis was performed using the SAS (version 9.3, SAS Institute Inc., Cary, NC, USA) software.

## 5. Conclusions

In conclusion, a significant proportion of unselected patients with pancreatic ductal adenocarcinoma carry clinically relevant germline pathogenic/likely pathogenic variants, irrespectively of age, family history or disease stage, while the median overall survival did not differ between pancreatic ductal adenocarcinoma carriers of pathogenic/likely pathogenic variants and non-carriers. In addition, no statistically significant benefit was observed from the administration of platinum-based therapy in patients carrying pathogenic/likely pathogenic variants in homologous recombination repair genes. Prospective studies evaluating the prognostic and predictive role of pathogenic/likely pathogenic variants through universal genetic testing of patients with pancreatic ductal adenocarcinoma, are warranted. 

## Figures and Tables

**Table 1:** Patient and tumor characteristics.

	Total (*N* = 549)	Pts with P/LPVs (*N* = 62)	Pts without P/LPVs (*N* = 487)	*p*-Value
**Age**				
Median (min, max)	65.0 (33.6, 86.0)	65.5 (41.8, 82.3)	65.0 (33.6, 86.0)	0.57 ^b^
**Sex**				0.67 ^c^
Female	271 (49.4)	29 (46.8)	242 (49.7)	
Male	278 (50.6)	33 (53.2)	245 (50.3)	
**Stage**				0.25 ^c^
Early	192 (35.0)	27 (43.5)	165 (33.9)	
Locally advanced	33 (6.0)	2 (3.2)	31 (6.4)	
Metastatic	324 (59.0)	33 (53.2)	291 (59.8)	
**Definitive surgery**				0.071
Yes	175 (31.9)	26 (41.9)	149 (30.6)	
No	374 (68.1)	36 (58.1)	338 (69.4)	
Histological grade *				0.32 ^c^
G1 (Well differentiated)	26 (6.0)	1 (2.0)	25 (6.5)	
G2 (Moderately differentiated)	215 (49.7)	25 (51.0)	190 (49.5)	
G3 (Poorly differentiated)	172 (39.7)	21 (42.9)	151 (39.3)	
G4 (Undifferentiated)	2 (0.46)	1 (2.0)	1 (0.26)	
GX (Grade cannot be assessed)	18 (4.2)	1 (2.0)	17 (4.4)	
Family history of cancer *				*0.011 ^c^*
No	330 (70.1)	33 (55.9)	297 (72.1)	
Yes	141 (29.9)	26 (44.1)	115 (27.9)	
Chemotherapy *				*0.037 ^c^*
No	9 (1.7)	3 (4.9)	6 (1.3)	
Yes	525 (98.3)	58 (95.1)	467 (98.7)	
**Platinum-based chemotherapy**				0.47 ^c^
No	275 (52.4)	33 (56.9)	242 (51.8)	
Yes	250 (47.6)	25 (43.1)	225 (48.2)	

* Data not available for all subjects. Missing values: Histological grade = 116, Family history of cancer = 78, Chemotherapy = 15. *p*-values: ^b^ = Wilcoxon rank-sum test, ^c^ = Pearson’s chi-square/Fisher’s exact test. Abbreviations: Pts: patients, P/LPV: pathogenic/likely pathogenic variant. Bold, italic values denote statistical significance at the 5% level of significance.

**Table 2:** Hazard ratios and 95% Confidence Intervals estimated by multivariate Cox regression analysis in the entire cohort with available data.

Parameter	Events/Total	HR (95% CI)	*p*-Value
**Stage**			
Early	118/181 (65.2%)	0.37 (0.29−0.46)	<0.001
Advanced	284/344 (82.6%)	Reference	--
**Interaction term of platinum-based CT with P/LPVs presence**			0.60
**P/LPVs among patients treated with platinum-based CT (*N* = 250)**			
Yes	18/25 (72%)	0.77 (0.47−1.23)	0.18
No	167/225 (74.2%)	Reference	--
**P/LPVs in non-platinum-based CT (*N* = 275)**			
Yes	20/33 (60.6%)	0.65 (0.43−1.05)	0.077
No	197/242 (81.4%)	Reference	--

Abbreviations: CI: confidence interval, CT: chemotherapy, HR: hazard ratio, N: number, P/LPV: pathogenic variant.

**Table 3:** Hazard ratios and 95% Confidence Intervals estimated by univariate Cox regression analysis upon exclusion of patients carrying only *MUTYH* P/LPVs and *CHEK2* p.Ile157Thr.

	Entire Cohort (*N* = 515)			Treated with Platinum-Based CT (*N* = 239)			No Platinum-Based CT (*N* = 262)		
	Events/Total	HR (95% CI)	*p*-Value	Events/Total	HR (95% CI)	*p*-Value	Events/Total	HR (95% CI)	*p*-Value
**Presence of P/LPVs**									
No	372/479 (77.7%)	Reference	--	167/225 (74.2%)	Reference	--	197/242 (81.4%)	Reference	--
Yes	23/36 (63.9%)	0.68 (0.44−1.03)	0.070	9/14 (64.3%)	0.58 (0.29−1.13)	0.11	12/20 (60%)	0.69 (0.39−1.24)	0.21
**P/LPVs in PDAC- associated genes**									
No	381/494 (77.1%)	Reference	--	172/232 (74.1%)	Reference	--	201/250 (80.4%)	Reference	--
Yes	14/21 (66.7%)	0.63 (0.37−1.08)	0.095	4/7 (57.1%)	0.34 (0.12−0.92)	0.033	8/12 (66.7%)	0.85 (0.42−1.73)	0.65
**P/LPVs in HRR genes**									
No	374/483 (77.4%)	Reference	--	168/226 (74.3%)	Reference	--	197/244 (80.7%)	Reference	--
Yes	21/32 (65.63%)	0.76 (0.49−1.18)	0.22	8/13 (61.5%)	0.52 (0.25−1.06)	0.074	12/18 (66.7%)	0.86 (0.48−1.55)	0.62

Abbreviations: CI: confidence interval, CT: chemotherapy, HR: hazard ratio, HRR: homologous recombination repair, N: number, PDAC: pancreatic ductal adenocarcinoma, P/LPV: pathogenic variant.

## Data Availability

The data presented in this study are available on request from the corresponding author. The data are not publicly available due to intellectual property reasons.

## Supplementary Materials

The following are available online at https://www.mdpi.com/2072-6694/13/2/198/s1, Supplementary methods, Figure S1: CNV-detection risk score plot for an ATM positive patient. Each dot denotes a targeted region with the y-axis representing the normalized read depth ratio (risk score) of the tested sample versus a set of normal samples. Horizontal dashed lines at y = 1.5 and y = 0.5 represent the expected risk score for a duplication and deletion, respectively, Table S1: Pathogenic/Likely pathogenic variants reported per patient, Table S2: P/LPV allele frequency analysis of low risk PVs in comparison with their frequency in the population (comparing against population groups with the highest frequency for each P/LPV).
