# Supplementary material for: Clinical Significance of Germline Cancer Predisposing Variants in Unselected Patients with Pancreatic Adenocarcinoma
Source: Cancers (Basel). 2021 Jan 8;13(2):198. doi: 10.3390/cancers13020198 (PMC7827324; doi:10.3390/cancers13020198)
Supplement: Supplementary file 1 [file cancers-13-00198-s001.pdf]

cancers

MDPI

Article

Clinical Significance of Germline Cancer Predisposing Variants in Unselected Patients with Pancreatic Adenocarcinoma

1. PreSENTIA™ pan-cancer panel

APC, ATM, BAP1, BARD1, BMPR1A, BRCA1, BRCA2, BRIP1, CDH1, CDK4, CDKN2A (CDKN2Ap16(INK4A), CDKN2Ap14(ARF)), CHEK2, DDB2, DICER1, EPCAM, ERCC1, ERCC2, ERCC3, ERCC4, ERCC5, FANCA, FANCB, FANCC, FANCD2, FANCE, FANCF, FANCG, FANCI, FANCL, FANCM, GREM1, HOXB13, MEN1, MLH1, MRE11, MSH2, MSH6, MUTYH, NBN, PALB2, PMS2, POLD1, POLE, POLH, PTEN, RAD50, RAD51C, RAD51D, RB1, RET, SDHAF2, SDHB, SDHC, SDHD, SLX4, SMAD4, SMARCA4, STK11, TP53, VHL, XPA, XPC.

2. Multigene testing

Genomic deoxyribonucleic acid (gDNA) was extracted using a standardized methodology and subjected to mechanical fragmentation prior to DNA library preparation. DNA libraries were then prepared based on previously established protocols.[1] DNA enrichment for the genomic regions of interest was carried out using a solution-based hybridization method with TACS (TArget Capture Sequences) (NIPD Genetics™). TACS were designed to capture selected loci in the genes of interest and were biotinylated after being generated by PCR. Biotinylated TACS were then immobilized on streptavidin coated magnetic beads for subsequent hybridization with the DNA libraries. Enriched DNA libraries were then normalized and were subjected to paired-end sequencing using manufacturer's protocols. PreSENTIA™ pan-cancer panel was used for the identification of SNVs, small Indels (≤20bp) and CNVs. All 549 germline DNA samples fulfilled the quality control criteria for SNV and Indel calling. Out of 549 germline DNA samples, 434 samples that their DNA quality was not affected by the sample's long-term storage were also subjected to CNV analysis using the abovementioned next-generation sequencing approach for all 62 genes.

3. Bioinformatics and Data Analysis

Sequencing data were de-multiplexed and aligned to the human genome build (hg19) using BWA-MEM to generate alignment (BAM) files. Specifically, for each sample, paired-end DNA sequencing reads were processed to remove adapter sequences and poor-quality reads. The remaining sequences were aligned to the human reference genome build hg19 using the Burrows-Wheeler alignment algorithm (BWA-MEM). Duplicate read entries were removed to convert aligned reads to a binary (BAM) file containing uniquely aligned read entries only. Per base allele-specific read-depth information was retrieved from this final BAM file. Variant calling was performed following GATK Best Practices workflow that implements local realignment and base quality score re-calibration.[2] Classification and interpretation of variants was conducted according to established guidelines provided by the American College of Medical Genetics and Genomics and the Association for Molecular Pathology[3] and was in line with ClinVar database.[4]

Germline CNV calling is performed using custom-build application programming interfaces (APIs) written in the Python and R programming languages (Python Software Foundation (2015) Python 2.7; The R Foundation (2015) The R Project for Statistical Computing v3.4.3). Pileup information on targeted bases is used to get probe-level read depth information. The statistical analysis for CNV detection at very high resolution is performed by a two-step (within and between samples) normalization method. The first step involves normalization of probe read depths using their expected value, which is estimated by fitting a local constant regression model on the probe read depths versus the

probe GC fraction. For each tested sample, an optimal subset of reference sample among a total of at least 100 reference samples is chosen to be used in the second normalization step. To this end, we developed a novel machine learning based method to identify said optimal subsets based on the enrichment similarity between the tested and reference samples and relies on principles of the k-nearest neighbors (KNN) machine learning classification algorithm. At this step, a subset of the reference samples is classified as similar to the tested sample and subsequently used as normalizers to get the final probe-level risk scores (Supplementary figure 1). Regions in the human genome containing repeats, pseudogenes and extreme GC-content have been excluded because they cannot be analyzed using short read sequencing. Each positive CNV or sequence variant call is confirmed with an orthogonal method.

P/LPV allele frequency analysis: Reference population frequency data were retrieved form the gnomAD population database containing both exome and genome sequencing data from a wide variety of large-scale sequencing projects.[5] The allele frequencies of the five most abundant P/LPVs in genes associated with low hereditary cancer risk (MUTYH, RAD50 and CHEK2) were compared against population groups with the highest frequency for each P/LPV using one-sided Fisher's exact test. Although this conservative approach lowers the risk for a type I error in our hypothesis testing, in the absence of multiple comparisons p-value correction (we have tested many genes at significance level 0.05), the results should only be treated as suggestive for further investigation. We did not apply a false discovery correction (this would render all p-values non-significant) since at this stage and given the design of the study (not a case-control study), we aimed at high sensitivity. By taking the conservative approach of testing against the population group with the highest frequency for each P/LPV, we maximized the probability of the true differences in frequencies to be statistically significant for our testing cohort even if its true frequency was lower than the one used.

Table S1. Pathogenic/Likely pathogenic variants reported per patient.

Patient ID	Transcript ID	Gene	CDS change	Amino acid change	Effect on RNA splicing pattern	Zygosity	ACMG Classification	ClinVar interpretation
PNC324	NM_000038.5	APC	c.2612delG	p.Gly871Glufs		Heterozygous	Pathogenic	Pathogenic
204116WB	NM_000051.3	ATM	c.8585-2A>C		abnormal splicing	Heterozygous	Pathogenic	Likely Pathogenic/Pathogenic
206983WB	NM_000051.3	ATM	c.67C>T	p.Arg23Ter		Heterozygous	Pathogenic	Pathogenic
207664WB	NM_000051.3	ATM	c.8766dupT	p.Val2923CysfsTer2		Heterozygous	Pathogenic	No ClinVar submission
207739WB	NM_000051.3	ATM	c.8585-2A>C		abnormal splicing	Heterozygous	Pathogenic	Likely Pathogenic/Pathogenic
207743WB	NM_000051.3	ATM	c.5979_5983delTAAAG	p.Ser1993ArgfsTer23		Heterozygous	Pathogenic	Pathogenic
213578BS	NM_000051.3	ATM	c.432dupA	p.Leu145ThrfsTer14		Heterozygous	Pathogenic	Likely Pathogenic/Pathogenic
PNC270	NM_000051.3	ATM	c.1215delT	p.Asn405LysfsTer15		Heterozygous	Pathogenic	Likely Pathogenic/Pathogenic
PNC354	NM_000051.3	ATM	exon2-4 deletion			Heterozygous	Pathogenic	Pathogenic
PNC436	NM_000051.3	ATM	c.2250G>A	p.Lys750=	abnormal splicing	Heterozygous	Pathogenic	Likely Pathogenic/Pathogenic
PNC534	NM_000051.3	ATM	c.1655delC	p.Pro552GlnfsTer4		Heterozygous	Pathogenic	Likely Pathogenic/Pathogenic
PNC538	NM_000051.3	ATM	c.5979_5983delTAAAG	p.Ser1993ArgfsTer23		Heterozygous	Pathogenic	Pathogenic
PNC234	NM_007294.3	BRCA1	c.329delA	p.Lys110ArgfsTer9		Heterozygous	Pathogenic	Pathogenic
PNC234	NM_001128425.1	MUTYH	c.734G>A	p.Arg245His		Heterozygous	Likely Pathogenic	Likely Pathogenic/Pathogenic
PNC323	NM_007294.3	BRCA1	c.5251C>T	p.Arg1751Ter		Heterozygous	Pathogenic	Pathogenic
207707WB	NM_000059.3	BRCA2	c.2644delC	p.Leu882PhefsTer13		Heterozygous	Pathogenic	Pathogenic
207745WB	NM_000059.3	BRCA2	c.9117G>A	p.Pro3039=	abnormal splicing	Heterozygous	Pathogenic	Pathogenic
PNC150	NM_000059.3	BRCA2	c.2339C>G	p.Ser780Ter		Heterozygous	Pathogenic	Pathogenic
PNC177	NM_000059.3	BRCA2	c.4284dupT	p.Gln1429SerfsTer9		Heterozygous	Pathogenic	Pathogenic
PNC311	NM_000059.3	BRCA2	c.2339C>G	p.Ser780Ter		Heterozygous	Pathogenic	Pathogenic

PNC393	NM_000059.3	BRCA2	c.9027delT	p.His3010IlefsTer18	Heterozygous	Pathogenic	Pathogenic
PNC186	NM_032043.2	BRIP1	c.633delT	p.Gly212AlafsTer62	Heterozygous	Pathogenic	Pathogenic
PNC523	NM_032043.2	BRIP1	c.2392C>T	p.Arg798Ter	Heterozygous	Pathogenic	Likely pathogenic/Pathogenic/Uncertain significance
204106WB	NM_007194.4	CHEK2	c.470T>C	p.Ile157Thr	Heterozygous	Pathogenic	Risk factor/Likely pathogenic/Pathogenic/Uncertain significance
205219WB	NM_007194.3	CHEK2	c.100C>T	p.Gln34Ter	Heterozygous	Pathogenic	Pathogenic
205219WB	NM_001128425.1	MUTYH	c.1187G>A	p.Gly396Asp	Heterozygous	Likely Pathogenic	Likely Pathogenic/Pathogenic
205255WB	NM_007194.4	CHEK2	c.470T>C	p.Ile157Thr	Heterozygous	Pathogenic	Risk factor/Likely pathogenic/Pathogenic/Uncertain significance
PNC215	NM_007194.4	CHEK2	c.470T>C	p.Ile157Thr	Heterozygous	Pathogenic	Risk factor/Likely pathogenic/Pathogenic/Uncertain significance
PNC216	NM_007194.4	CHEK2	c.470T>C	p.Ile157Thr	Heterozygous	Pathogenic	Risk factor/Likely pathogenic/Pathogenic/Uncertain significance
PNC244	NM_007194.4	CHEK2	c.470T>C	p.Ile157Thr	Heterozygous	Pathogenic	Risk factor/Likely pathogenic/Pathogenic/Uncertain significance
PNC244	NM_001128425.1	MUTYH	c.1187G>A	p.Gly396Asp	Heterozygous	Likely Pathogenic	Likely Pathogenic/Pathogenic
PNC461	NM_007194.4	CHEK2	c.470T>C	p.Ile157Thr	Heterozygous	Pathogenic	Risk factor/Likely pathogenic/Pathogenic/Uncertain significance

PNC469	NM_007194.4	CHEK2	c.470T>C	p.Ile157Thr	Heterozygous	Pathogenic	Risk factor/Likely pathogenic/Pathogenic/Uncertain significance
PNC49	NM_007194.4	CHEK2	c.470T>C	p.Ile157Thr	Heterozygous	Pathogenic	Risk factor/Likely pathogenic/Pathogenic/Uncertain significance
PNC513	NM_007194.4	CHEK2	c.470T>C	p.Ile157Thr	Heterozygous	Pathogenic	Risk factor/Likely pathogenic/Pathogenic/Uncertain significance
207609WB	NM_000122.1	ERCC3	c.325C>T	p.Arg109Ter	Heterozygous	Pathogenic	Likely Pathogenic/Pathogenic
PNC151	NM_000136.2	FANCC	c.455dupA	p.Asn152LysfsTer9	Heterozygous	Pathogenic	Likely Pathogenic/Pathogenic
PNC541	NM_000136.2	FANCC	exon2-3 deletion		Heterozygous	Pathogenic	Pathogenic
PNC165	NM_020937.3	FANCM	c.5101C>T	p.Gln1701Ter	Heterozygous	Pathogenic	Likely Pathogenic/Pathogenic/Risk factor
207533WB	NM_000179.2	MSH6	c.3514dupA	p.Arg1172LysfsTer5	Heterozygous	Pathogenic	Pathogenic
205212WB	NM_001128425.1	MUTYH	c.734G>A	p.Arg245His	Heterozygous	Likely Pathogenic	Likely Pathogenic/Pathogenic
205250WB	NM_001128425.1	MUTYH	c.734G>A	p.Arg245His	Heterozygous	Likely Pathogenic	Likely Pathogenic/Pathogenic
207605WB	NM_001128425.1	MUTYH	c.734G>A	p.Arg245His	Heterozygous	Likely Pathogenic	Likely Pathogenic/Pathogenic
207744WB	NM_001128425.1	MUTYH	c.734G>A	p.Arg245His	Heterozygous	Likely Pathogenic	Likely Pathogenic/Pathogenic
207854WB	NM_001128425.1	MUTYH	c.1187G>A	p.Gly396Asp	Heterozygous	Likely Pathogenic	Likely Pathogenic/Pathogenic
PNC126	NM_001128425.1	MUTYH	c.734G>A	p.Arg245His	Heterozygous	Likely Pathogenic	Likely Pathogenic/Pathogenic
PNC145	NM_001128425.1	MUTYH	c.734G>A	p.Arg245His	Heterozygous	Likely Pathogenic	Likely Pathogenic/Pathogenic

PNC166	NM_001128425.1	MUTYH	c.1437_1439delGGA	p.Glu480del		Heterozygous	Pathogenic	Pathogenic
PNC230	NM_001128425.1	MUTYH	c.1171C>T	p.Gln391Ter		Heterozygous	Pathogenic	Pathogenic
PNC264	NM_001128425.1	MUTYH	c.504+19_504+31delTA GGGGAAATAGG		abnormal splicing	Heterozygous	Likely Patho-genic	Likely Patho-genic/Pathogenic
PNC288	NM_001128425.1	MUTYH	c.536A>G	p.Tyr179Cys		Heterozygous	Pathogenic	Likely Patho-genic/Pathogenic
PNC319	NM_001128425.1	MUTYH	c.734G>A	p.Arg245His		Heterozygous	Likely Patho-genic	Likely Patho-genic/Pathogenic
PNC326	NM_001128425.1	MUTYH	c.1437_1439delGGA	p.Glu480del		Heterozygous	Pathogenic	Pathogenic
PNC364	NM_001128425.1	MUTYH	c.1187G>A	p.Gly396Asp		Heterozygous	Likely Patho-genic	Likely Patho-genic/Pathogenic
PNC392	NM_001128425.1	MUTYH	c.734G>A	p.Arg245His		Heterozygous	Likely Patho-genic	Likely Patho-genic/Pathogenic
PNC550	NM_001128425.1	MUTYH	c.1437_1439delGGA	p.Glu480del		Heterozygous	Pathogenic	Pathogenic
207527WB	NM_002485.4	NBN	c.657_661delACAAA	p.Lys219AsnfsTer16		Heterozygous	Pathogenic	Pathogenic
PNC487	NM_024675.3	PALB2	c.2257C>T	p.Arg753Ter		Heterozygous	Pathogenic	Pathogenic
207013WB	NM_005732.3	RAD50	c.326_329delCAGA	p.Thr109AsnfsTer20		Heterozygous	Pathogenic	Likely Patho-genic/Pathogenic
207528WB	NM_005732.3	RAD50	c.326_329delCAGA	p.Thr109AsnfsTer20		Heterozygous	Pathogenic	Likely Patho-genic/Pathogenic
PNC182	NM_005732.3	RAD50	c.326_329delCAGA	p.Thr109AsnfsTer20		Heterozygous	Pathogenic	Likely Patho-genic/Pathogenic
PNC481	NM_005732.3	RAD50	c.326_329delCAGA	p.Thr109AsnfsTer20		Heterozygous	Pathogenic	Likely Patho-genic/Pathogenic
PNC528	NM_058216.2	RAD51C	c.904+5G>T		abnormal splicing	Heterozygous	Likely Patho-genic	Likely Pathogenic
PNC549	NM_058216.2	RAD51C	c.181_182delCT	p.Leu61AlafsTer11		Heterozygous	Pathogenic	Pathogenic
PNC525	NM_003000.2	SDHB	c.445C>T	p.Gln149Ter		Heterozygous	Pathogenic	Pathogenic

Table S2. P/LPV allele frequency analysis of low-risk PVs in comparison with their frequency in the population (comparing against population groups with the highest frequency for each P/LPV).

Gene	Mutation	AA change	Patient cohort			Population frequency			Fisher test p-value
			Number of PV alleles	Total alleles	PV allele frequency	PV allele frequency in population group with the highest fraction	Allele count	Allele number	
MUTYH	**C/T**	**p.Arg245His**	9	1098	0.008197	0.001166 (Bulgarian)	3	2572	1.69E-03
CHEK2	A/G	p.Ile157Thr	9	1098	0.008197	0.04594 (Estonian)	222	4832	1.00E+00
RAD50	A/-4	p.Thr109AsnfsTer20	5	1098	0.004554	0.002624 (Bulgarian)	7	2668	2.55E-01
MUTYH	T/-3	p.Glu480del	3	1098	0.002732	0.0006085 (Southern European)	7	11504	4.9E-02
MUTYH	C/T	p.Gly396Asp	4	1098	0.003643	0.006378 (North-western European)	322	50490	9.17E-01

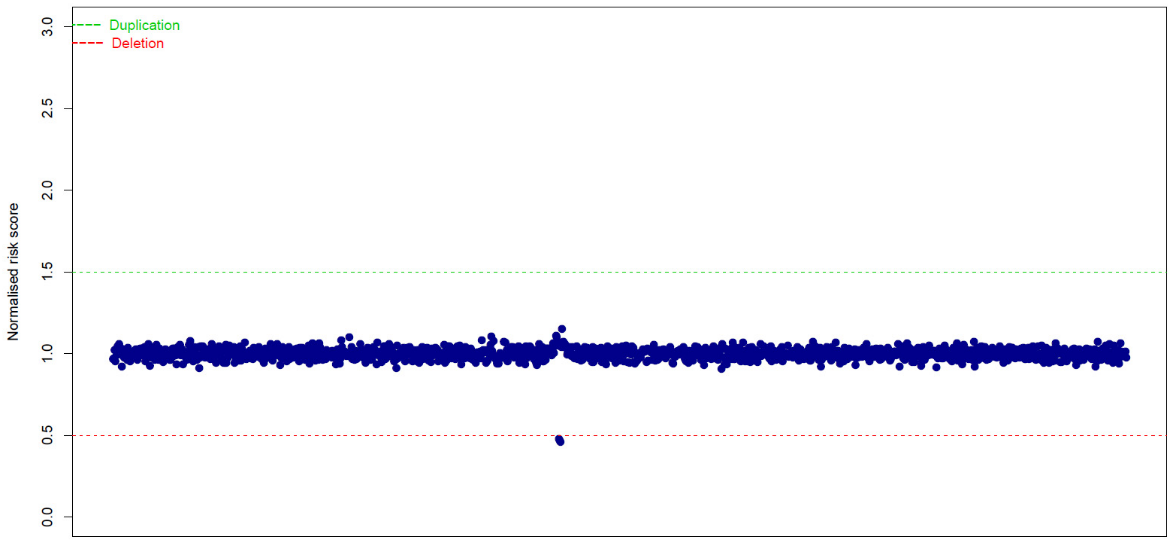

Figure S1. CNV-detection risk score plot for an ATM positive patient. Each dot denotes a targeted region with the y-axis representing the normalized read depth ratio (risk score) of the tested sample versus a set of normal samples. Horizontal dashed lines at y=1.5 and y=0.5 represent the expected risk score for a duplication and deletion, respectively.

References

1. Koumbaris, G.; Kypri, E.; Tsangaras, K.; Achilleos, A.; Mina, P.; Neofytou, M.; Velissariou, V.; Christopoulou, G.; Kallikas, I.; González-Liñán, A.; et al. Cell-Free DNA Analysis of Targeted Genomic Regions in Maternal Plasma for Non-Invasive Prenatal Testing of Trisomy 21, Trisomy 18, Trisomy 13, and Fetal Sex. *Clin. Chem.* **2016**, *62*, 848–855, doi:10.1373/clinchem.2015.252502.
2. McKenna, A.; Hanna, M.; Banks, E.; Sivachenko, A.; Cibulskis, K.; Kernytsky, A.; Garimella, K.; Altshuler, D.; Gabriel, S.B.; Daly, M.J.; et al. The Genome Analysis Toolkit: A MapReduce framework for analyzing next-generation DNA sequencing data. *Genome Res.* **2010**, *20*, 1297–1303, doi:10.1101/gr.107524.110.
3. Richards, S.; Aziz, N.; Bale, S.; Bick, D.; Das, S.; Gastier-Foster, J.; Grody, W.W.; Hegde, M.; Lyon, E.; Spector, E.; et al. Standards and guidelines for the interpretation of sequence variants: a joint consensus recommendation of the American College of Medical Genetics and Genomics and the Association for Molecular Pathology. *Genet. Med.* **2015**, *17*, 405–423, doi:10.1038/gim.2015.30.
4. Landrum, M.J.; Lee, J.M.; Benson, M.; Brown, G.R.; Chao, C.; Chitipiralla, S.; Gu, B.; Hart, J.; Hoffman, D.; Jang, W.; et al. ClinVar: improving access to variant interpretations and supporting evidence. *Nucleic Acids Res.* **2018**, *46*, D1062–D1067, doi:10.1093/nar/gkx1153.
5. Karczewski KJ, Francioli LC, Tiao G, et al: Variation across 141,456 human exomes and genomes reveals the spectrum of loss-of-function intolerance across human protein-coding genes. bioRxiv:531210, 2019.
